# Insects in anthelminthics research: Lady beetle-derived harmonine affects survival, reproduction and stem cell proliferation of *Schistosoma mansoni*

**DOI:** 10.1371/journal.pntd.0007240

**Published:** 2019-03-14

**Authors:** Josina Kellershohn, Laura Thomas, Steffen R. Hahnel, Arnold Grünweller, Roland K. Hartmann, Martin Hardt, Andreas Vilcinskas, Christoph G. Grevelding, Simone Haeberlein

**Affiliations:** 1 Institute of Parasitology, BFS, Justus Liebig University, Giessen, Germany; 2 Institute of Pharmaceutical Chemistry, Philipps University, Marburg, Germany; 3 Biomedical Research Center Seltersberg—Imaging Unit, Justus Liebig University, Giessen, Germany; 4 Institute for Insect Biotechnology, Justus Liebig University, Giessen, Germany; University of Würzburg, GERMANY

## Abstract

Natural products have moved into the spotlight as possible sources for new drugs in the treatment of helminth infections including schistosomiasis. Surprisingly, insect-derived compounds have largely been neglected so far in the search for novel anthelminthics, despite the generally recognized high potential of insect biotechnology for drug discovery. This motivated us to assess the antischistosomal capacity of harmonine, an antimicrobial alkaloid from the harlequin ladybird *Harmonia axyridis* that raised high interest in insect biotechnology in recent years. We observed remarkably pleiotropic effects of harmonine on physiological, cellular, and molecular processes in adult male and female *Schistosoma mansoni* at concentrations as low as 5 μM *in vitro*. This included tegumental damage, gut dilatation, dysplasia of gonads, a complete stop of egg production at 10 μM, and increased production of abnormally shaped eggs at 5 μM. Motility was reduced with an EC50 of 8.8 μM and lethal effects occurred at 10–20 μM within 3 days of culture. Enzyme inhibition assays revealed acetylcholinesterase (AChE) as one potential target of harmonine. To assess possible effects on stem cells, which represent attractive anthelminthic targets, we developed a novel *in silico* 3D reconstruction of gonads based on confocal laser scanning microscopy of worms after EdU incorporation to allow for quantification of proliferating stem cells per organ. Harmonine significantly reduced the number of proliferating stem cells in testes, ovaries, and also the number of proliferating parenchymal neoblasts. This was further supported by a downregulated expression of the stem cell markers *nanos-1* and *nanos-2* in harmonine-treated worms revealed by quantitative real-time PCR. Our data demonstrate a multifaceted antischistosomal activity of the lady beetle-derived compound harmonine, and suggest AChE and stem cell genes as possible targets. Harmonine is the first animal-derived alkaloid detected to have antischistosomal capacity. This study highlights the potential of exploiting insects as a source for the discovery of anthelminthics.

## Author summary

Natural compounds represent one of the richest sources for the discovery of new active compounds against diseases such as cancer or infections, including helminth infections that cause the highest disease burden in tropical countries. Surprisingly, insects have been almost completely neglected with respect to anthelminthics discovery although they represent the most species-rich class of animals known on earth, producing a wide spectrum of compounds with biological activities. In insect biotechnology, the harlequin ladybird *Harmonia axyridis* raised high interest being a rich source of antimicrobial compounds such as the alkaloid harmonine. Harmonine is thought to act as a chemical weapon keeping otherwise detrimental microsporidia in the beetle under control. Testing the antiparasitic potential of harmonine against adult *Schistosoma mansoni*, one of the most harmful helminths worldwide, resulted in multifaceted negative effects. The compound damaged tissues essential for survival and reproduction of schistosomes (tegument, intestine, gonads) and also affected stem-cell proliferation. Furthermore, we obtained first evidence for acetylcholinesterase as one potential molecular target, which was partially inhibited by harmonine. This is the first time to proof a direct effect of a defined insect-derived compound on a helminth parasite, a finding that will encourage further studies to explore insects as sources of novel anthelminthics.

## Introduction

Natural compounds represent one of the richest sources for the discovery of new active compounds against cancer, infections, or other threats to human health. From 1981 to 2010, 33% of approved drugs represented natural compounds and derivatives, mostly from plants, algae, and fungi [1]. In recent years, the search for novel anthelmintic compounds from natural sources has been intensified with the aim to identify new hit and lead compounds for drug development [2]. So far, medicinal plants and their metabolites (like alkaloids, terpenes, and peptides) have been widely exploited as sources of novel natural compounds with anthelmintic activity. In contrast, only few studies have focused on animal-derived molecules [3]. Surprisingly, although insects are among the most successful and widespread organisms on earth, especially regarding their diversity and adaptability, they are still rather underrated as sources of compounds with medical importance. Along these lines, insects have been almost completely neglected with respect to anthelminthic discovery [4].

The chemical defense of insects against pathogens and parasites relies on effector molecules such as antimicrobial peptides and secondary metabolites. The invasive harlequin ladybird *Harmonia axyridis*, which is also known as Asian ladybird or Multicolored ladybird, represents an outstanding example in this regard [5]. Its immune system encompasses more than fifty antimicrobial peptides, the highest number ever reported for an animal [6]. In addition, its hemolymph contains extraordinarily high concentrations of the constitutively expressed antimicrobial alkaloid harmonine ((17R,9Z)‐1,17‐diaminooctadec‐9‐ene), an aliphatic, long-chain diamine which displays antimicrobial activities [7]. Its superior immune system promotes its invasive success in a multifaceted manner. The beetle’s antimicrobial peptides have been demonstrated to mediate resistance against pathogenic bacteria [8], whereas harmonine has been postulated to keep its microsporidia under control. Microsporidia are highly specialized relatives of fungi that propagate as intracellular parasites in insects and other taxa [9]. *H*. *axyridis* carries a high load of microsporidia which can infect and kill native competitors such as the two-spotted ladybird *Adalia bipunctata* and the seven-spotted ladybird *Coccinella septempunctata* when transmitted e.g. during intraguild predation. Therefore, these parasites have been postulated to function in invaded areas like bioweapons to successfully outcompete native competitors [6, 10, 11]. In addition, first tests against human pathogens showed that harmonine displays a broad-spectrum antimicrobial activity including *in vitro* effects against mycobacteria as well as protozoan parasites [7, 12]. Activity against helminths has so far not been investigated, even though novel active compounds are highly needed for a whole list of helminth infections, which includes neglected tropical diseases (NTDs) in humans and veterinary infectious diseases [13, 14].

Schistosomiasis is among the helminthic diseases causing highest disability and mortality in humans worldwide [15]. In endemic areas, schistosomiasis occurs as a chronic disease, which derives from the longevity of blood-resident adult schistosomes persisting many years in the host. Pathology is mainly caused by tissue deposition of eggs which are produced in hundreds per day by the female worm [16]. Approximately 700 million people are at risk of schistosomiasis [15], and there is no vaccination that might prevent infection. Treatment relies on a single drug, praziquantel (PZQ). PZQ is active against all three major schistosome species, *Schistosoma mansoni*, *S*. *haematobium* and *S*. *japonicum*, it can be produced at low cost and is well tolerated. It is therefore used in mass treatment programs and as preventive chemotherapy for people at high risk [17]. This and its continued use since the 1980s have increased the risk of resistance development. Indeed, evidence was obtained for schistosomes with lowered PZQ susceptibility in human drug administration programs and in experimental animal models [18–20]. Therefore, finding alternative treatment options has become an urgent issue [21].

Due to their outstanding species number and diversity, insects constitute a huge “drug cabinet” to be explored which might also include novel compounds with anthelmintic activity. To make a start, we focused in our study on the antimicrobial alkaloid compound harmonine derived from the harlequin ladybird. The aim of this study was to reveal whether this insect-derived compound shows anthelmintic capacity against adult *S*. *mansoni* worms. By physiological, cellular, and molecular analyses we observed a complex multifaceted phenotype comprising tegumental damage, gut dilatation, gonadal dysplasia, egg-production deficits as well as cellular and molecular effects on stem cells. Furthermore, we found first evidence for AChE as one potential target. With the results of our study, we want to promote insect-derived compounds and move them into spot-light as possible sources of new drugs in the treatment of schistosomiasis and further parasitic diseases.

## Materials & methods

### Ethics statement

Animal experiments were performed in accordance with the European Convention for the Protection of Vertebrate Animals used for experimental and other scientific purposes (ETS No 123; revised Appendix A) and have been approved by the Regional Council (Regierungspraesidium) Giessen (V54-19 c 20/15 c h 02 GI 18/10Nr. A 1/2014).

### Parasites

A Liberian strain (Bayer AG, Monheim) of *S*. *mansoni* was used to infect freshwater snails of the genus *Biomphalaria glabrata* as intermediate host and Syrian hamsters (*Mesocricetus auratus*) as final host [22, 23]. Eight week-old hamsters were obtained from Janvier (France), infected by the “paddling method” [24], and sacrificed at 46 days p.i. to collect adult worm couples by perfusion. Unisexual worm populations were generated by monomiracidial intermediate-host infection [23]. Worms were cultured in M199 medium (Sigma-Aldrich, Germany; supplemented with 10% Newborn Calf Serum (NCS), 1% HEPES [1 M] and 1% ABAM-solution [10,000 units penicillin, 10 mg streptomycin and 25 mg amphotericin B per ml]) at 37°C and 5% CO_2_.

### *In vitro* culture

For *in vitro* culture of adult couples with harmonine, worms were cultured in 6-well plates in supplemented M199 medium with 10 worm couples per well. Harmonine was synthesized as described by Nagel et al. 2015 [12] and kindly provided by W. Boland (Max-Planck-Institute for Chemical Ecology, Jena, Germany). Harmonine was dissolved in DMSO and added in final concentrations of 2.5–50 μM as indicated. As negative control, M199 medium was adjusted to the same concentration of DMSO as used for the highest inhibitor concentration. The worms were incubated at 37°C and 5% CO_2_ for 72 h, medium and harmonine were exchanged every 24 h. Harmonine-induced morphological effects were assessed every 24 h using an inverted microscope (Leica, Germany). Worm motility was scored with a system following recommendations by WHO-TDR [25], with the scores 3 (normal motility), 2 (reduced motility), 1 (minimal and sporadic movements), 0 (no movements within 30 sec was considered dead). For depletion of proliferating cells, 20 mM hydroxyurea was added to the culture for 72 h, and medium plus hydroxyurea was refreshed every 24 h. For visualization of proliferating cells, EdU (5-ethynyl-2-deoxyuridine) was added to a final concentration of 10 mM for the last 24 h of *in vitro* culture. Thereafter, worms were either processed for confocal laser scanning microscopy (CLSM), or subjected to RNA extraction for quantitative real-time PCR (qPCR) analysis.

### Quantitative real-time PCR

Freshly perfused or *in vitro*-cultured worm couples were separated by gender, and RNA was extracted from male and female worms using the PeqGOLD TriFast reagent (Peqlab, Germany) according to the manufacturer’s protocol. RNA quality was checked using the Agilent RNA 6000 Nano kit and an Agilent Bioanalyzer 2100 instrument (Agilent Technologies, USA), followed by reverse transcription using the Quantitect RT-Kit (Qiagen, Germany). Expression levels of the *S*. *mansoni* orthologs of the stem cell markers *nanos-1* (Smp_055740) and *nanos-2* (Smp_051920), and the AChE ortholog (Smp_154600) were determined by qPCR using the SYBR Green method [26] with the PerfeCTa SYBR Green SuperMix (VWR, Germany), the Rotor-Gene Q instrument and Rotor-Gene Q Series Software (Qiagen). All samples were pipetted in technical triplicates. Ct values were normalized against the geometric mean of three references genes selected based on stable expression in both sexes (Haeberlein et al., submitted): orthologs of LETM1 (Smp_065110), phosphatase 2A (Smp_166290) and proteasome-beta (Smp_073410). Relative expression levels were calculated either by the delta delta Ct method [27] or by expressing the data as n-fold difference by the formula: relative expression = 2^−delta Ct^ × f, with f = 100 as an arbitrary factor (as indicated in the figure legends). The following primers were used, which were confirmed by test qPCRs to have efficacies between 0.9–1: LETM1_fw 5’-GAAGGTGATCAAGCTCCATTGT-3’, LETM1_rev 5’-TTGTACTGCATGGATAGGTGGT-3’; phosphatase-2A_fw 5’-GTAAAACTGGTCCATTTGAAGAAC-3’, phosphatase-2A_rev 5’-TACCGAATAGGAAATGTTGAACGA-3’; prot-beta_fw 5’-GGTCTGGTGGTTTCTCGTTC-3’, prot-beta_rev 5’-GTACCTTCTGTTGCCCGTG-3’; nanos-1_fw 5’-ACTTGTCCATTATGCGGTGCT-3’, nanos-1_rev 5’- GGTTCCAACAAACCAGCTTCA-3’; nanos-2_fw 5’-GCCGTGTTATGACCTCTGG-3’, nanos-2_rev 5’-GACGATCTGGAGACTCTGG-3’; AChE1_fw 5’-GATGATGATGATGAACGACCG-3’, AChE1_rev 5’- CAGTAACTAATGATTATCGTATACCA-3’; AChE2_fw 5’-TAAGACACGAAATGATGATTCACG-3’, AChE2_rev 5’-TACTTCATATTGTGTAGTTGATTGAC-3’.

### Protein isolation

Native protein lysates were prepared from 50 male or 150 female worms as described [28]. Briefly, worms were sonicated in PBS supplemented with protease inhibitors. The protein concentration was determined by the advanced protein assay reagent (APAR, Cytoskeleton Inc., USA) and measured at 590 nm in a microplate reader (VARIOSKAN FLASH; Thermo Fisher Scientific, USA).

### Acetylcholinesterase assay

AChE activity was determined in native protein lysates of adult male or female *S*. *mansoni* using the Amplite Colorimetric Acetylcholinesterase Assay Kit (AAT Bioquest, Biomol, Germany) following the instruction of the manufacturer, which is based on Ellman’s method [29]. For inhibition studies, the protocol was adapted as follows: for each harmonine concentration, 200 μg/ml of native male protein or 100 mU/ml of the AChE standard (from the electric eel *Electrophorus electricus*) were added. A negative control containing an equivalent amount of solvent of harmonine (DMSO) was included. To start the reaction, an AChE reaction mixture was added to each well. The final concentration of the substrate acetylthiocholine was 0.5 mM. Absorbance by the reaction product TNB-thiocholine, which is proportional to the AChE activity, was measured by the VARIOSKAN FLASH microplate reader at 405 nm with reads every 10 min at 37°C.

### Confocal laser scanning microscopy

For morphological analysis by CLSM, worms were fixed and stained with carmine red (CertistainH; Merck, Germany) as described before [30, 31]. For EdU labelling and detection of proliferating cells, the Click-iT Plus EdU Alexa Fluor 488 Imaging Kit (Thermo Fisher Scientific) was used. After 24 h of incubation with EdU, couples were separated, fixed and stained as described [32]. Worms were counterstained with Hoechst 33342 in a final concentration of 8 μM. Stained worms were examined on an inverse CLSM (Leica TSC SP5; Leica, Germany). Hoechst was excited with a 405 nm laser, and Alexafluor488 as well as carmine red with an argon-ion laser at 488 nm. Laser power as well as gain and offset of all photomultiplier tubes (PMTs) were optimized for minimizing possible bleaching effects and for full range intensity coding using the CLUT-function (color look-up table) of the Leica LAS AF software. Background signals and optical section thickness were defined by setting the pinhole size to airy unit 1. The software package “IMARIS for cell biologists” (Bitplane, Switzerland) was used to quantify Hoechst- and EdU-positive cells in ovaries and testes of worms. Z-stacks acquired by CLSM were used as input data. First, a so-called surface was created manually for each organ to extract it *in silico* from the surrounding tissue. Next, surfaces were created for all EdU- and Hoechst-positive cells. This allowed quantifying stained cells per organ. To minimize counting of artifacts or background noise, a threshold was set prior to surface creations that excluded objects <3 μm.

### Statistical analysis

Statistical analysis was performed using an unpaired t-test. A *p*-value < 0.05 was considered significant.

## Results

### Harmonine reduces schistosome motility and viability *in vitro*

**Fig 1:**
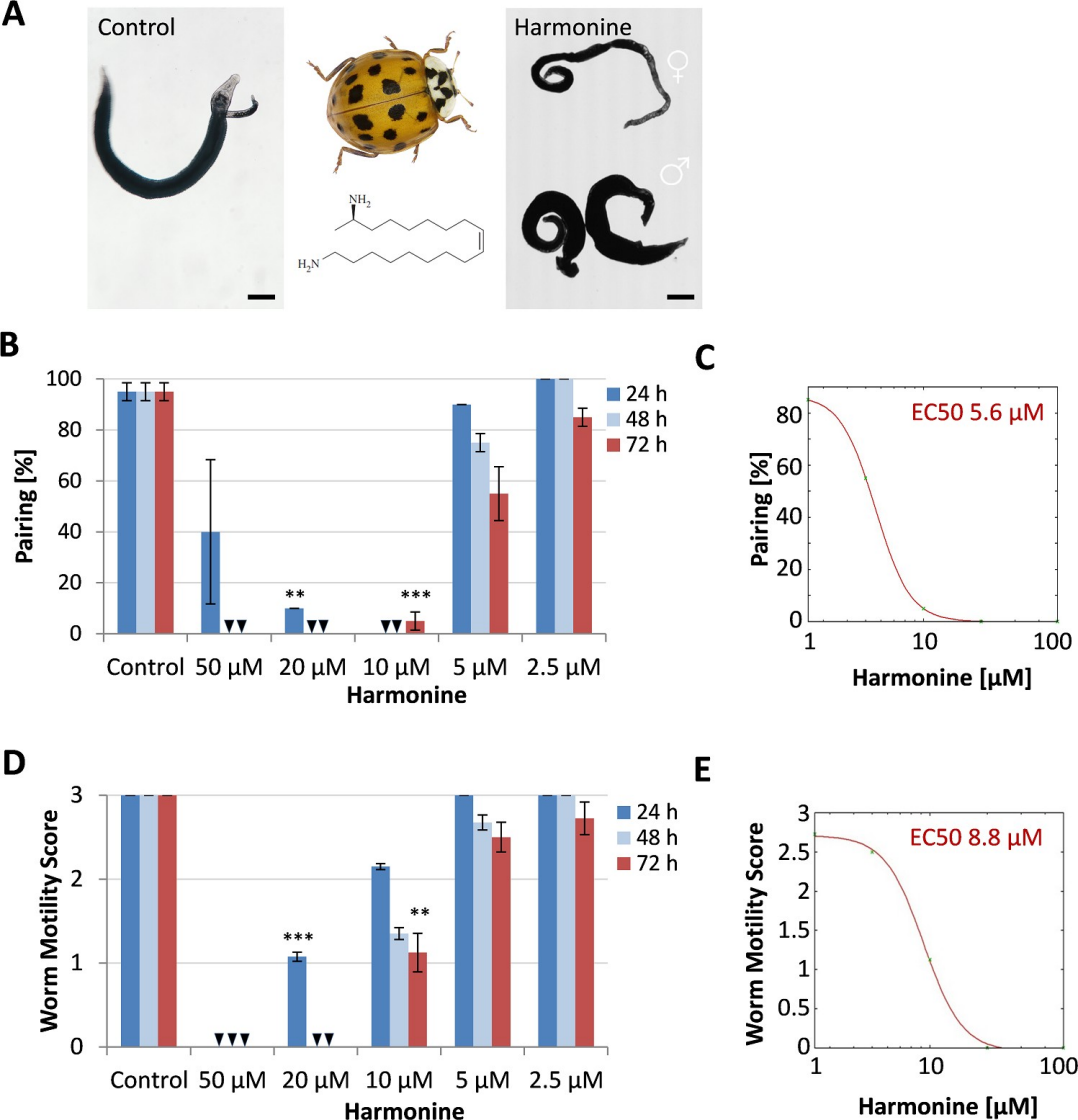
Effect of harmonine on motility, viability and pairing. *S*. *mansoni* couples were treated with different concentrations of harmonine for 72 h and microscopically screened every 24 h. As a negative control, the solvent DMSO was added in an amount as present in the highest harmonine concentration. **(A)** Representative images of a control couple and worms after treatment with 10 μM harmonine for 72 h. Scale bar: 100 μm. *H*. *axyridis* [33] and the structure of the alkaloid harmonine are shown in the middle. **(B)** Percentage of paired worms after harmonine treatment. **(C)** Dose-response curve for pairing stability (expressed as % pairing) after 72 h of treatment. **(D)** Worm motility reflected by motility scores 3 = normal motility, 2 = reduced motility, 1 = almost no movements, 0 = dead. **(E)** Dose-response curve for the reduction of motility by harmonine after 72 h of treatment. EC50 values were calculated by non-linear least squares curve fitting using the ic50.tk tool. A-C show a summary of two experiments with 10 worm couples per experiment and condition; error bars: SEM. Triangles indicate a value of zero. Significant differences to control worms at the respective time point are indicated with ** *p*<0.01 or *** *p*<0.001 (students t-test).

**Fig 2:**
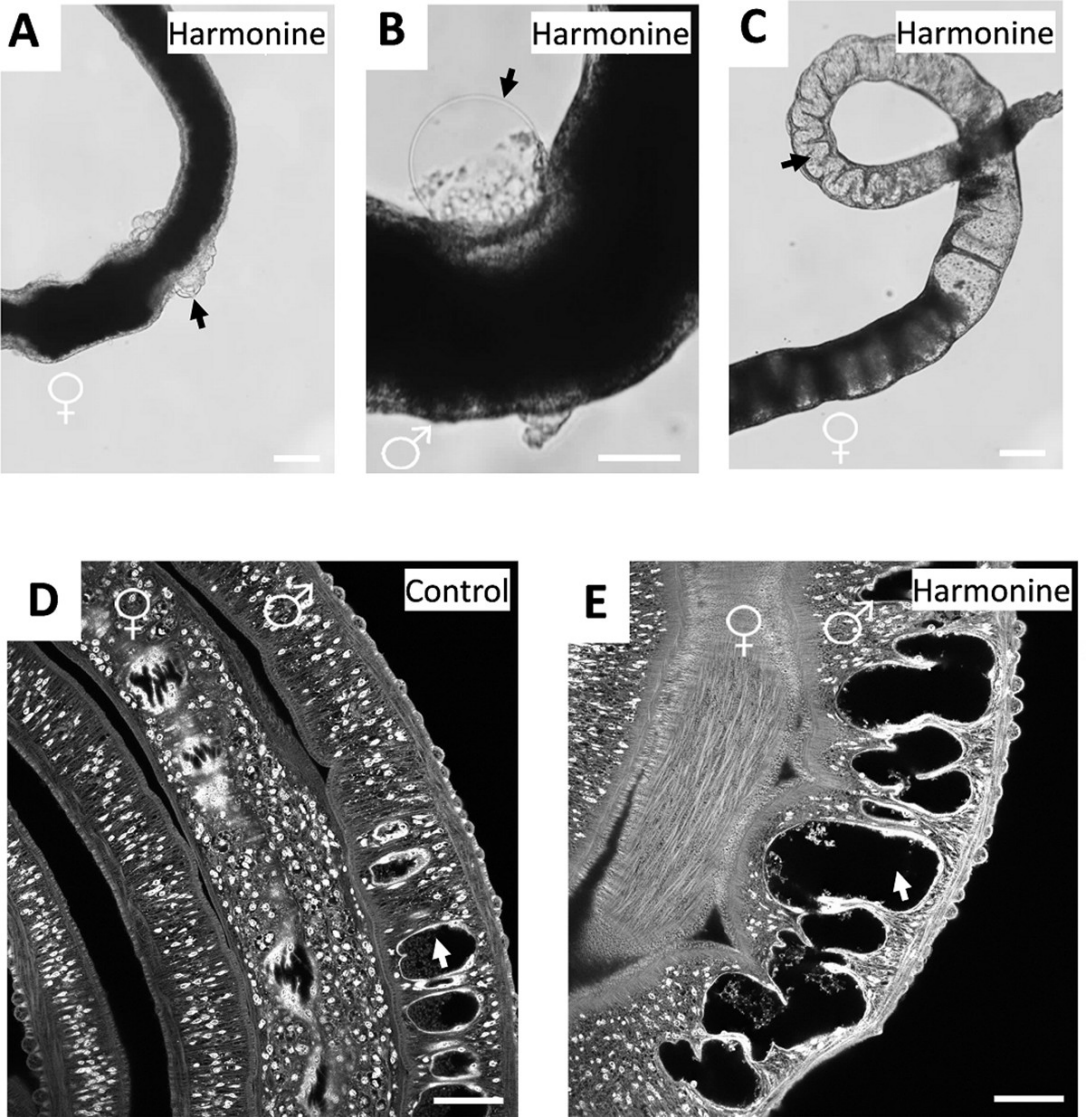
Tissue damage induced by harmonine. *S*. *mansoni* couples were cultured for 72 h with 5 μM harmonine with a change of medium and compound every 24 h. **(A, B)** Bubble formation (arrows) on the tegument surface in a representative female (A) and male (B) worm. **(C)** Gut dilatation (arrow) in a female worm, visualized by bright-field microscopy. **(D, E)** CLSM images of carmine red-stained worms showing the gut lumen of a control male (D) and the dilated lumen (arrow) of a harmonine-treated male (E). Scale bars are 100 μm (A-C) or 50 μm (D, E).

To investigate whether the insect-derived compound harmonine has anthelminthic activity, adult *S*. *mansoni* worms were cultured *in vitro* with different concentrations of the compound over a period of 72 h. Harmonine reduced the pairing stability of worm couples in a dose-dependent manner (Fig 1A–1C). With ≥ 10 μM harmonine, all couples separated within 48 h and were detached from the culture-well surface. With 5 μM, around 50% of worm couples separated (calculated EC50: 5.6 μM), and 90% were detached after 72 h. A similar time- and dose-dependent pattern was found for the reduction of worm motility by harmonine (Fig 1D), with a calculated EC50 of 8.8 μM (Fig 1E). Specifically, with concentrations of 50 and 20 μM, all worms died within 2 h and 48 h, respectively. With 10 μM, worms had a significantly reduced motility with an average motility score of 1 after 48–72 h, indicating only minimal and sporadic movements; 25% of worms were dead after 72 h. Interestingly, most of the females were unaffected with 10 μM after the first day, whereas treated males showed reduced motility, indicating a gender bias in the effect of harmonine. Concentrations below 10 μM caused a weak, time-dependent reduction of motility. Next to its effects on motility, viability and pairing stability, harmonine affected worm tissue structure in remarkable ways. Concentrations as low as 5 μM induced bubble formation on the tegumental surface in both males and females (Fig 2A and 2B). In addition, severe gut dilatations were observed in both sexes of *S*. *mansoni* by bright-field microscopy, which was confirmed by CLSM (Fig 2C–2E). Taken together, harmonine showed antischistosomal activity and induced a dose-dependent spectrum of phenotypic effects in schistosomes, ranging from tissue damage and pairing-instability with 5 μM, severe impairment of worm motility up to death at 10 μM, and finally 100% lethality at 20 μM.

### Harmonine reduces acetylcholinesterase activity

**Fig 3:**
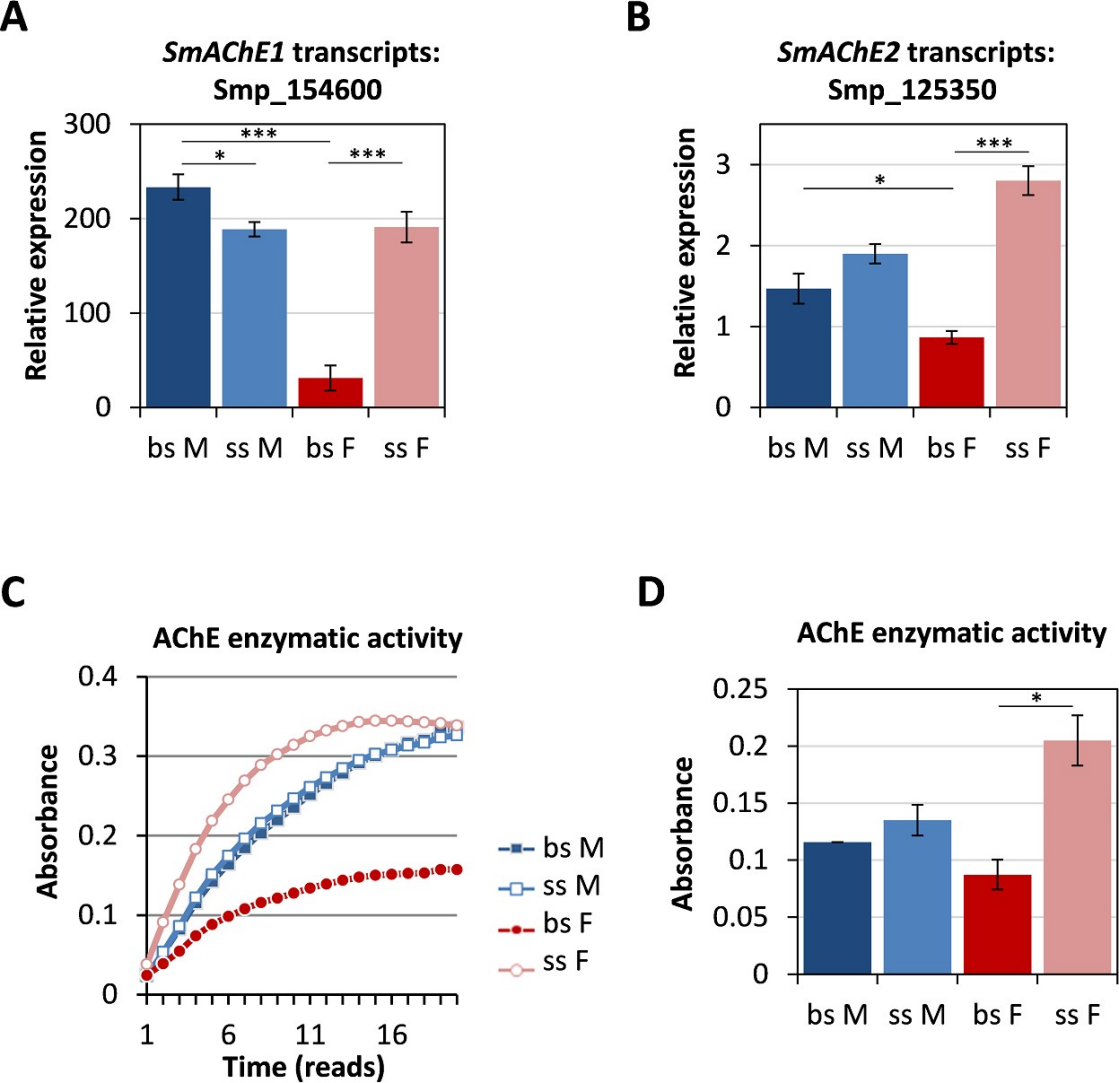
AChE transcript levels in *S*. *mansoni* and inhibition of enzymatic activity by harmonine. **(A, B)** Expression of *SmAChE1* (Smp_154600) and *SmAChE2* (Smp_125350) in different sexes and mating-states as determined by qPCR, expressed as relative expression vs. the geometric mean of three reference genes (x100 as an arbitrary factor). A summary of three experiments with SEM is shown. **(C, D)** Enzymatic activity present in protein lysates of male and female worms in the paired or unpaired state given as change of absorbance over time of one representative of two experiments (C) and as summary of two experiments (absorbance at 30 min) (D). Enzymatic activity was determined by Ellman’s method by quantifying absorbance of 5-thio-2-nitrobenzoic acid, which is equivalent to the amount of thiocholine produced from the hydrolysis of acetylthiocholine by AChE. Significant differences as determined by the unpaired t-test are indicated with * *p*<0.05 or *** *p*<0.001. M, male; F, female; bs, bisex (paired); ss, single-sex (unpaired).

Reduced motility, pairing-instability and gut dilatations might point to a target of harmonine involved in (neuro)muscular activity. In addition, the observed tegumental effects suggested target localization within the tegument. Previous *in silico* docking analyses with *Leishmania* proteins suggested AChE as a possible target of harmonine [34]. AChE is a well-characterized enzyme, also in schistosomes, and it is the presumed target of metrifonate, a formerly used antischistosomal drug [35]. AChE was found to be abundantly expressed in the tegument of schistosomes [36, 37]. Therefore, we hypothesized that AChE might be one target of harmonine in *S*. *mansoni*. By SMART analysis of protein sequences from genes electronically annotated as AChEs in GeneDB and WormBase ParaSite, we identified two potential orthologs of AChE in *S*. *mansoni*: Smp_125350 and Smp_154600, which both show the typical carboxyl-esterase domain (S1 Fig). We suggest the name *SmAChE1* for Smp_154600 because of its first characterization in a previous study [38], and *SmAChE2* for Smp_125350. Based on preliminary data of a transcriptomics study [39], we first characterized the expression levels of both *SmAChE* genes in the different sexes as well as the enzymatic activity of their protein lysates. AChE transcript levels were determined by qPCR and revealed a sex- and pairing-dependent expression (Fig 3A and 3B). Notably, expression in females after pairing contact (from bisexual infection, bs F) was significantly decreased compared to females in unpaired state (from single-sex infection, ss F) or males. For females, this difference in transcript level correlated well with enzyme activity since we determined a significantly lower AChE enzymatic activity in protein lysates of paired females compared to unpaired females. A similar trend was observed for paired females vs. males (Fig 3C and 3D).

**Fig 4:**
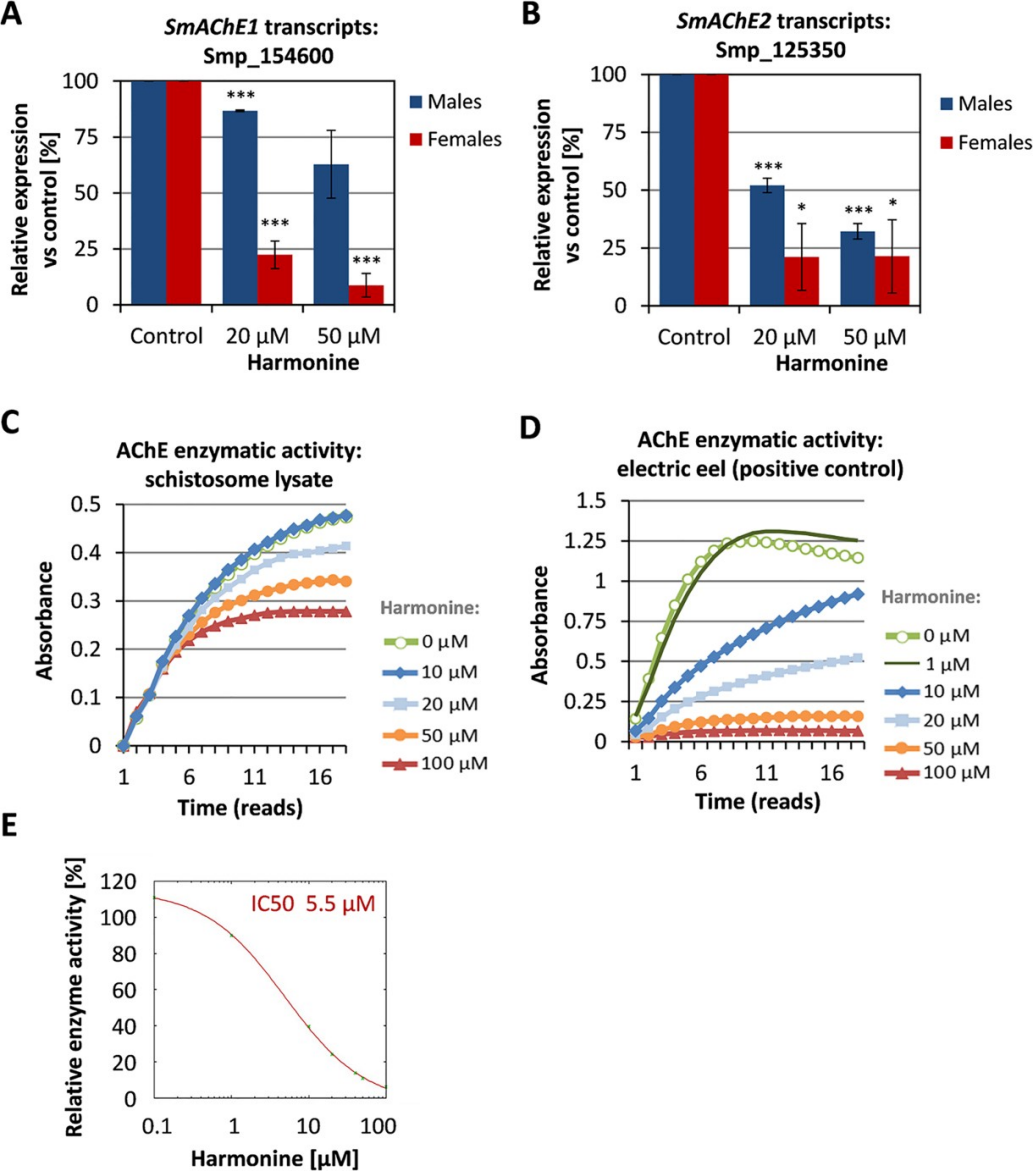
Inhibition of AChE enzymatic activity by harmonine. **(A, B)** Expression of *SmAChE1* (Smp_154600) and *SmAChE2* (Smp_125350) in male and female schistosomes after 72 h treatment with different concentrations of harmonine as determined by qPCR, expressed as relative expression vs. the geometric mean of three reference genes. The expression in control worms was set to 100%. A summary of two experiments with SEM is shown. **(C)** Enzymatic activity over time in protein lysates of paired males after adding different concentrations of harmonine (0 – 100 μM). One representative out of three similar experiments is shown. **(D)** Enzymatic activity of AChE from a model organism (electric eel) after addition of different concentrations of harmonine (0 – 100 μM). One representative out of two similar experiments is shown. **(E)** Relative AChE activity of electric eel at 30 min after adding different concentrations of harmonine, with the activity at 0 μM set as 100%. Mean values of two experiments were used. IC50 was calculated by non-linear least squares curve fitting using the ic50.tk tool. Significant differences as determined by the unpaired t-test are indicated with * *p*<0.05 or *** *p*<0.001.

Next we investigated a possible effect of harmonine on AChE transcript levels. The expression of both genes was reduced in a dose-dependent manner in harmonine-treated compared to control male and female worms (Fig 4A and 4B). Because of the low AChE activity in protein lysates of paired females, male lysates were used to test the capacity of harmonine for inhibiting schistosomal AChE activity. Harmonine (10 – 100 μM) decreased the turnover of the substrate acetylthiocholine in a dose-dependent manner (Fig 4C). Interestingly, the inhibition of AChE from a common test organism (electric eel) [40] was even more efficient, and it occurred at lower concentrations (Fig 4D). With an IC50 of 5.5 μM against electric eel AChE (Fig 4E), harmonine can be considered a moderate inhibitor of AChE activity, whose potency might be species-dependent. Also compared to IC50 values of the known AChE inhibitor physostigmine (electric eel, 148.4 nM; schistosome lysate, 724.2 nM; S2A–S2D Fig), harmonine was clearly less potent. As increasing excess concentrations of the substrate ACh at constant harmonine concentration did not restore full AChE reaction velocity, an inhibition mechanism other than competitive inhibition is likely (S2E Fig).

### Harmonine affects reproduction and gonadal tissues

**Fig 5:**
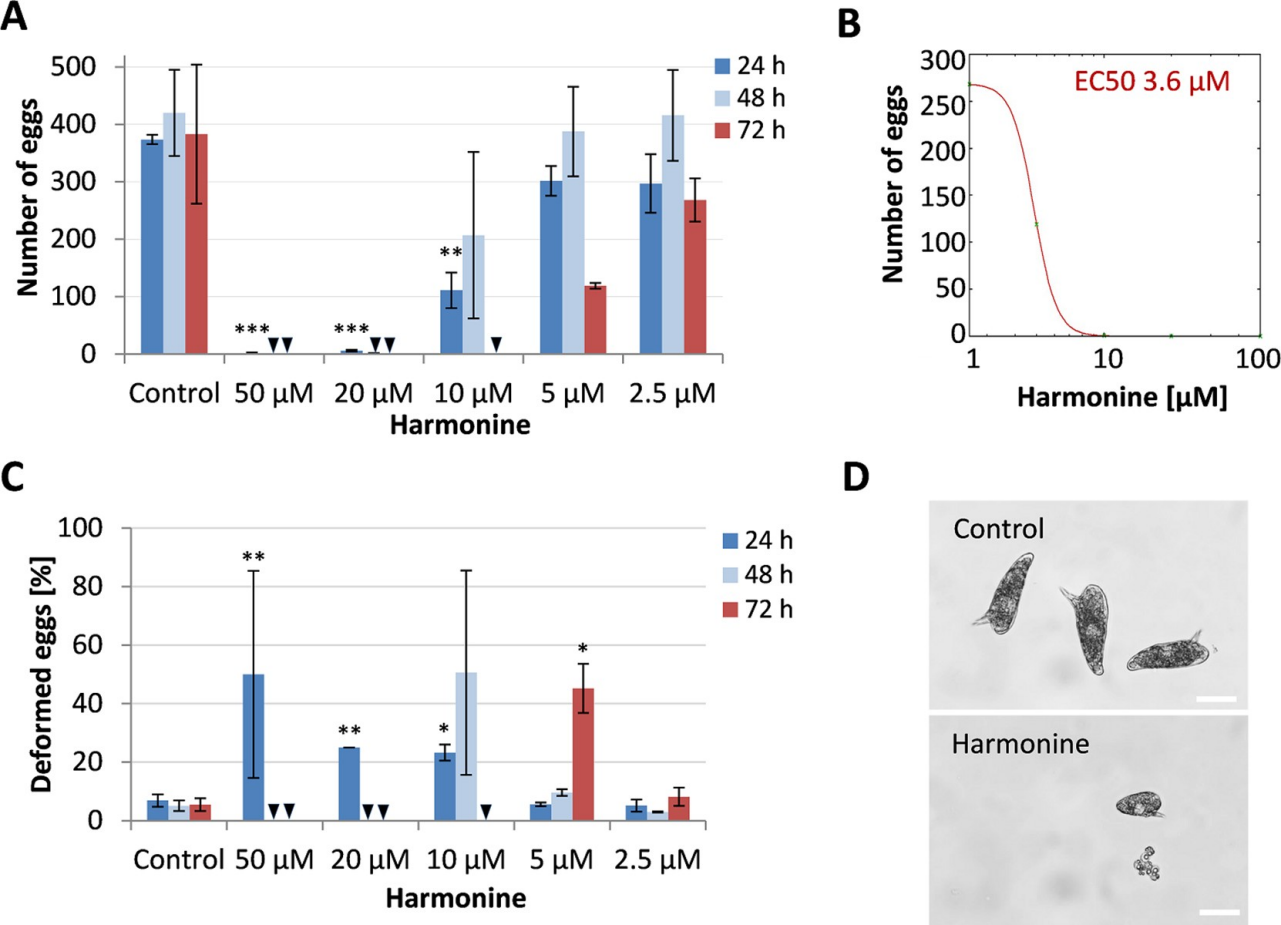
Effect of harmonine on egg production. *S*. *mansoni* couples were cultured with different concentrations of harmonine for a period of 72 h. Medium and compound were renewed every 24 h. **(A, C)** Effect of harmonine on reproduction was assessed by counting the number of total eggs being laid by 10 couples per 24 h period (A) and their percentage of deformed eggs (C). **(B)** Dose-response curve for the reduction of egg numbers being laid in the last 24 h of the 72 h culture. Summary of two experiments with 10 couples of *S*. *mansoni* per experiment and condition; error bars: SEM. **(D)** Representative images of eggs from control couples and after 72 h treatment with 5 μM harmonine. Deformed eggs were e.g. smaller and often lacked an oocyte, a spine, or a normal amount of vitelline cells. Scale bar is 50 μm. Significant differences to the control at the respective time point are indicated with * *p*<0.05, ** *p*<0.01 or *** *p*<0.001 (students t-test).

Besides motility and morphology, compound-induced effects on reproduction are also of high interest because schistosome eggs are essential for maintaining the life-cycle and causative for the pathology of schistosomiasis. We therefore determined the quantity and quality of egg production during 72 h treatment of adult worm couples with harmonine. Egg production ceased completely with 20 μM harmonine after 48 h and with 10 μM after 72 h, respectively (Fig 5A). 5 μM harmonine reduced the number of eggs to 31% compared to the control, but of note, up to 57% of these eggs were of abnormal size and shape. In addition, free vitellocytes were found in the culture medium indicating egg-production deficits (Fig 5C and 5D). Overall, the EC50 of harmonine for the reduction of egg production was 3.6 μM (Fig 5B).

**Fig 6:**
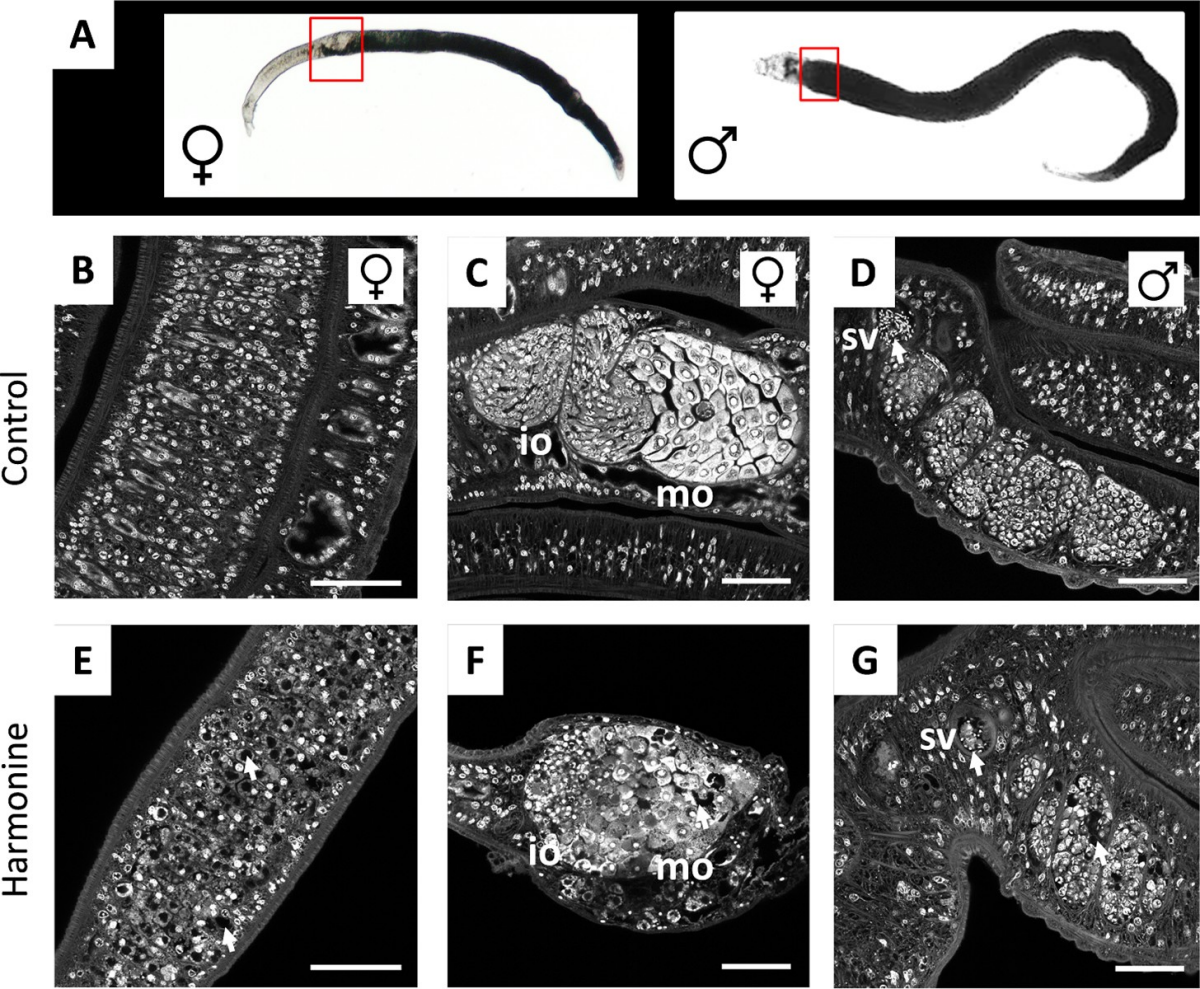
Effect of harmonine on gonadal tissue structure. For CLSM-analysis of gonadal tissues, *S*. *mansoni* couples were cultured for 72 h with 10 μM harmonine (E-G) or solvent as a control (B-D), and stained with carmine red. **(A)** Bright-field microscopic images indicating the localization of ovary and testes in worms. **(B, E)** Confocal images showing part of the intact vitellarium of a control female (still paired with a male) (B) compared to the porous appearance of the vitellarium after harmonine treatment (E). **(C, F)** Well-defined immature (iO) and mature (mO) parts of a control ovary (C), compared to the disintegrated structure of an ovary in a harmonine-treated female (F). **(D, G)** Seminal vesicle (sv) filled with spermatozoa, and testes lobes filled with spermatogonia of a control male (D), compared with the gonad of a harmonine-treated male with reduced number of spermatozoa and partially disintegrated lobes (arrows) (G). Scale bar: 50 μm.

To investigate whether impaired egg production was related to gonadal tissue defects, harmonine-treated worms were stained with carmine red for subsequent CLSM analysis, which allowed the detection of morphologic abnormalities at the organ level. As expected [30, 31], in control females the vitellarium was found to be tightly packed with cells, and it was arranged similar to a zipper with cell rows interlocking with the opposite side (Fig 6B). Treatment with harmonine at a concentration of 10 μM led to the formation of numerous unstained, hole-like areas, giving the whole vitellarium a *swiss cheese*-*like* tissue pattern (Fig 6E). In the ovary of control females, the small immature oogonia are located within the anterior part and the bigger, mature oocytes within the larger posterior part (Fig 6A and 6C), as shown before [30, 31]. Already at 10 μM, harmonine clearly disrupted the ordered structure of the ovary (Fig 6F). The ovary appeared shrunken, oogonia of smaller size and mature oocytes poorly separated from each other. The cytoplasm stained weaker compared to control ovaries, and unstained, hole-like areas were found similar to the phenotype seen in the vitellarium.

In control males, testes consist of distinct lobes which are tightly packed with spermatogonia [30, 31]. At the anterior end, mature spermatocytes gather in the seminal vesicle (Fig 6D). After harmonine treatment, lobes also appeared shrunken and showed similar porous areas as seen in female tissues. The seminal vesicle contained less spermatocytes and an undefined cell mass instead, which probably represents undifferentiated spermatogonia (Fig 6G). All observed phenotypes were occasionally observed also after treatment with 5 μM harmonine. To sum up, treatment with harmonine led to a dramatic reduction and malformation of schistosomal eggs, which might be related to the structural disruption of ovary, vitellarium, and testes.

### Defects in gonadal stem cell proliferation

**Fig 7:**
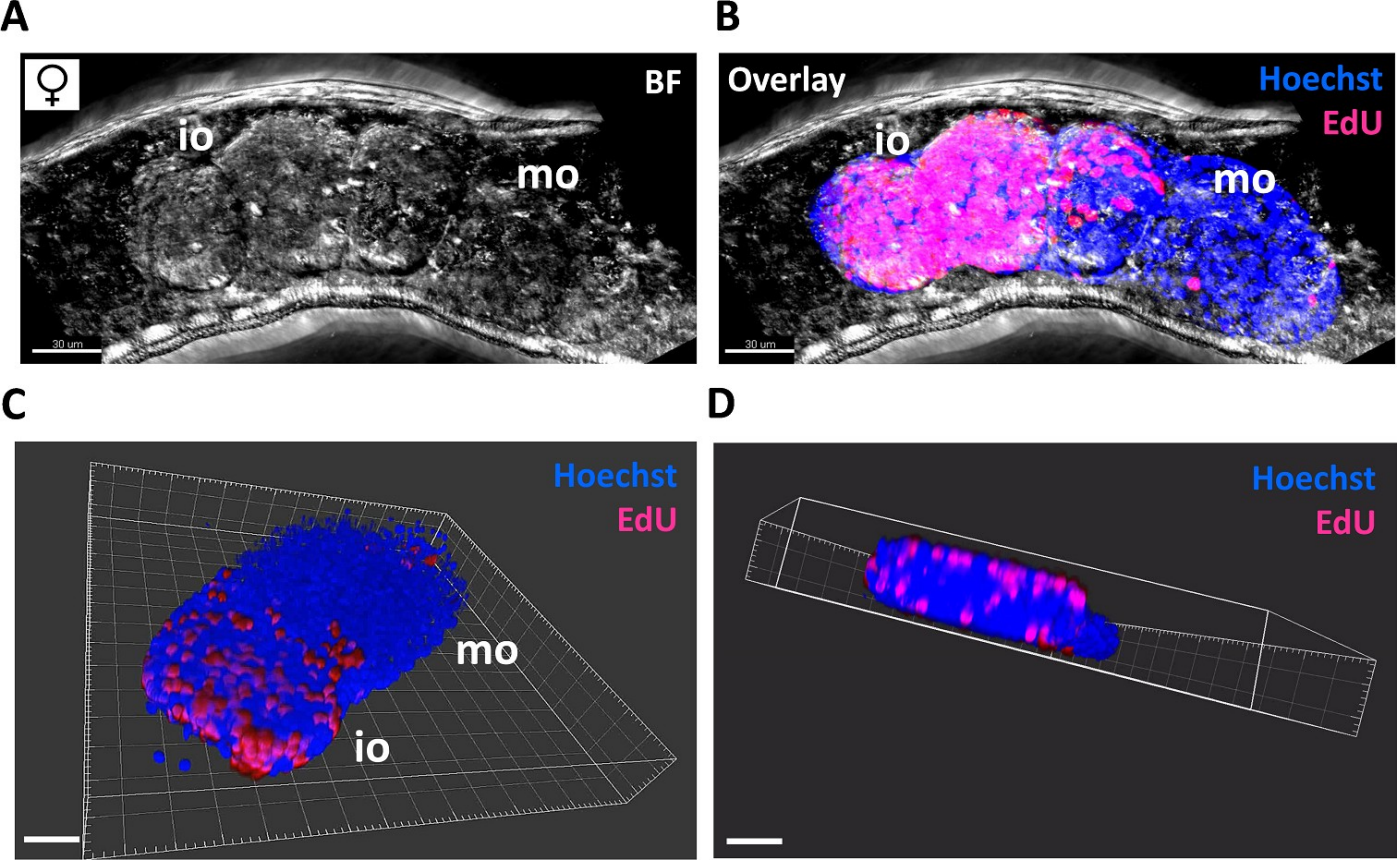
3D reconstruction of *S*. *mansoni* ovary and stem cell localization. Couples were cultured for 24 h with EdU. Females were separated from males, stained with Hoechst, and z-stacks of the ovary and surrounding tissue were acquired by CLSM. Z-stacks were processed with the IMARIS software to release the ovary from the surrounding tissue and to quantify the number of EdU- and Hoechst-positive cells. **(A)** Representative z-stack showing the immature part (iO) and mature part (mO) of an ovary within a female worm in bright field (BF). **(B)** The same z-stack with an overlay of the BF image and the *in silico*-released ovary. Proliferating, EdU-positive stem cells are depicted in pink, Hoechst-positive cells in blue. **(C, D)** Still images of a video animation of a 3D reconstructed ovary (see supplementary video, S1 Movie). (D) shows the preferential localization of stem cells at the edges of the immature part of the ovary. Scale bar: 30 μm (A, B) or 40 μm (C, D).

**Fig 8:**
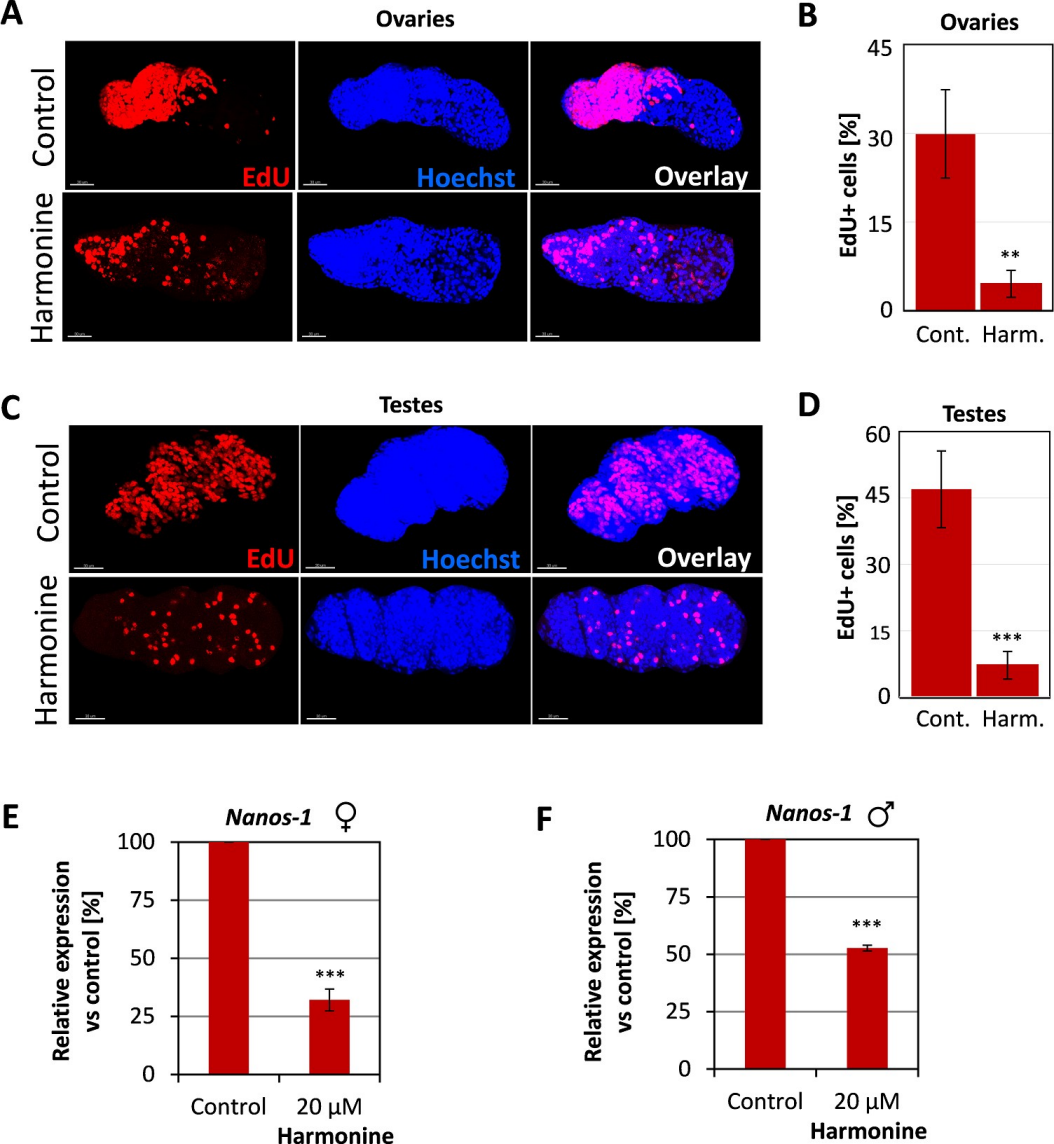
Reduction of gonadal stem cell proliferation upon harmonine treatment. *S*. *mansoni* couples were treated with 20 μM harmonine *in vitro* for 6 days. As control, an equivalent volume of the solvent DMSO was used. EdU was added for the last 24 h of culture, worm couples were separated and images were processed using the IMARIS software as described in the text and Fig 6. **(A, B)** Representative images (A) and summary of the analyses of four ovaries (B) from either control or harmonine-treated females. A significant reduction of stem cells was found after treatment, expressed as percentage of EdU-positive per Hoechst-positive cells. **(C, D)** Representative images (C) and summary of four testes (C) from control and harmonine-treated males showing a significant reduction of the percentage of stem cells following treatment. Scale bar: 30 μm. **(E, F)** Expression of the gonadal stem cell marker *nanos-1* (Smp_055740) in females (E) and males (F) after treatment with 20 μM harmonine compared to control worms as determined by qPCR. The expression in control worms was set to 100%. Summary of two experiments. An unpaired t-test was performed to reveal significant differences (** *p*<0.01, *** *p*<0.001).

Two findings led to the hypothesis that harmonine might affect stem cell proliferation. First, one of the non-neuronal functions of AChE described for humans is related to stem-cell activity [41]. Second, the observed harmonine-induced impairment of gonadal cell organization might point to a defect in a preceding step, i.e. gonadal stem-cell proliferation. Therefore, we used the thymidine analogue EdU to visualize proliferating cells in schistosomal tissues of harmonine-treated worms vs. controls by CLSM. As background staining we used Hoechst. Indeed, sublethal concentrations of 5 and 10 μM harmonine reduced the number of proliferating cells in both ovaries and testes compared to organs of control worms (S3 Fig). In order to quantify the number of proliferating stem cells per organ as an objective measure, we established a procedure to separate gonads *in silico* from the surrounding worm tissue with the help of the analysis software IMARIS. 3D visualization of ovaries revealed that proliferating stem cells are exclusively located in the anterior part (Fig 7). In addition, stem cells are not homogeneously nested between oogonia, but preferentially located at the outer edge of the organ (Fig 7C and 7D; S1 Movie). Next, we determined the percentage of EdU-positive stem cells per total Hoechst-positive cells for each ovary or testis. The percentage of proliferating stem cells per organ was significantly reduced in harmonine-treated worms compared to control worms. While control ovaries and testes showed on average 30% and 47% EdU-positive cells, respectively, the percentage dropped to 5% and 7% after exposure to harmonine (Fig 8A–8D). This resulted in a merely scattered distribution of stem cells within the gonads. To support these findings, we investigated whether harmonine might also affect the transcript level of *nanos-1* (Smp_055740), a gene described as germline-specific stem cell marker in adult *S*. *mansoni* [42, 43]. Indeed, the transcript level of *nanos-1* was significantly reduced in harmonine-treated male and female worms (Fig 8E and 8F).

### Impairment of neoblast proliferation

**Fig 9:**
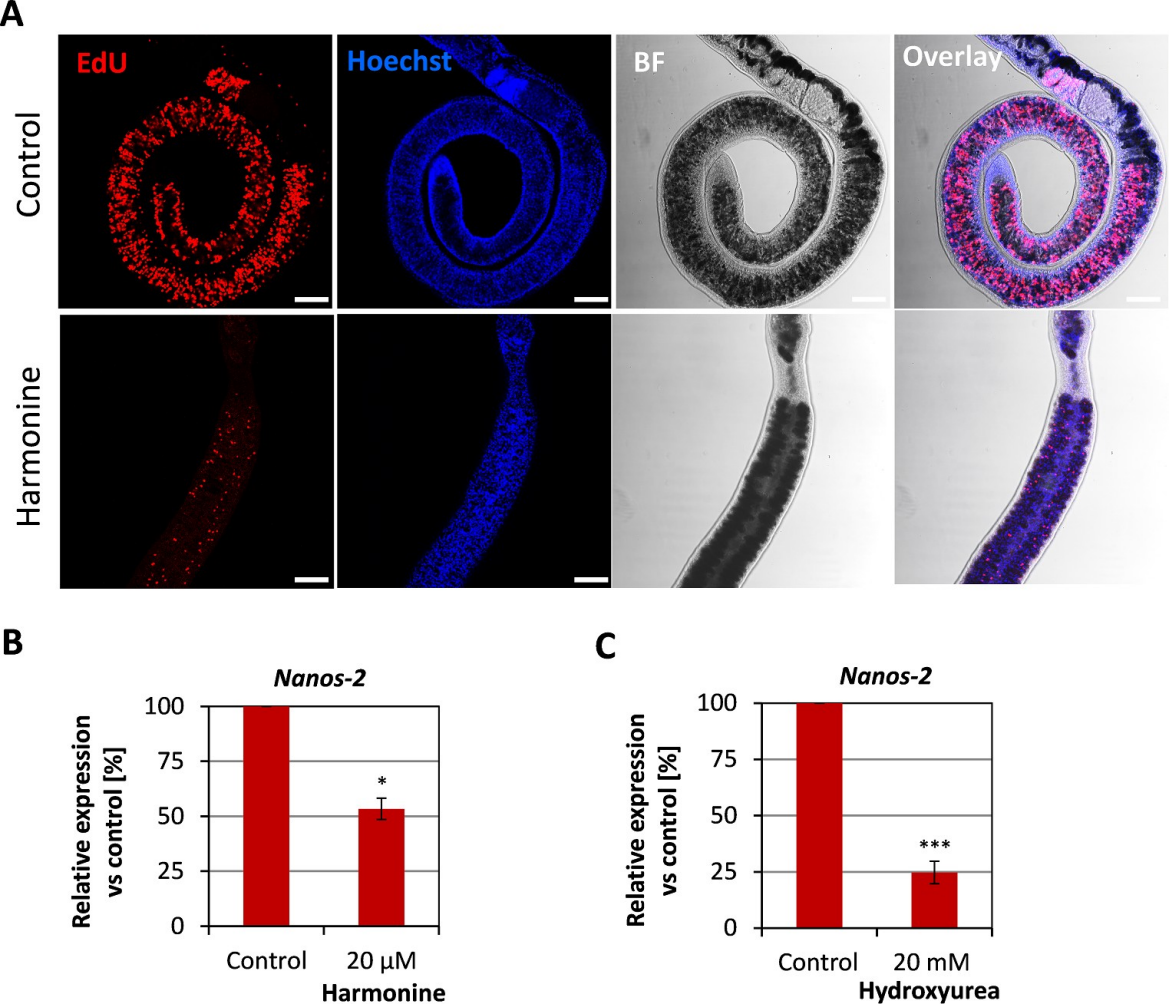
Reduction in the number of proliferating neoblasts upon harmonine treatment. *S*. *mansoni* couples were treated and processed for EdU labeling of proliferating cells as described in Fig 7. **(A)** Representative images of a control and a harmonine-treated female showing proliferating cells (neoblasts) in the parenchyma and/or vitellarium. Scale bar: 100 μm. BF, bright field. **(B, C)** Expression of the stem cell marker *nanos-2* (Smp_051920) in harmonine-treated (B) or hydroxyurea-treated (C) females related to the expression in untreated control females (set to 100%) as determined by qPCR (summary of two experiments). An unpaired t-test was performed to reveal significant differences (* *p*<0.05, *** *p*<0.001).

In addition to the gonads, proliferating stem cell-like cells are also present in the parenchyma of *S*. *mansoni* [44]. These so-called neoblasts are thought to provide replenishment of tegumental and gastrodermal cells. Impairment of these cells by a compound like harmonine would therefore be a useful way to interfere with worm survival. Similar to proliferating cells in the reproductive organs, the amount of EdU-positive cells decreased after treatment with harmonine in males and females. In the representative control female depicted in Fig 9A (top, left image), a huge number of EdU-positive cells was also detected in the vitellarium, while after treatment, only some residual cells were found (Fig 9A, bottom, left image).

*Nanos-2* (Smp_051920) is a stem cell marker expressed both in germline and somatic stem cells. *Nanos-2* transcripts were found to be reduced after irradiation-mediated depletion of somatic stem cells [44]. We therefore used it as a molecular measure for compound-induced effects on neoblasts. *Nanos-2* transcript levels were determined by qPCR after treatment of worms with harmonine or hydroxyurea for comparison. Hydroxyurea is a mitotic inhibitor that was successfully used before to deplete the majority of neoblasts in helminths [45, 46]. Compared to control worms, the expression of *nanos-2* was significantly decreased by almost 50% after treatment with 20 μM harmonine (Fig 9B). Hydroxyurea reduced expression fourfold at a concentration of 20 mM (Fig 9C).

Taken together, harmonine reduced the number of proliferating stem cells, both gonadal and parenchymal ones, in *S*. *mansoni*, which was proven by gonad-specific quantification of EdU-positive cells and a reduced expression of the stem cell markers *nanos-1* and *nanos-2*.

## Discussion

The aim of the study was to assess the antischistosomal capacity and possible targets of harmonine, an antimicrobial compound from the harlequin ladybird *Harmonia axyridis* which attracted increasing interest due to its antimicrobial activities [7]. Our results demonstrate a wide spectrum of effects by harmonine on *S*. *mansoni* adults, including (1) reduction of motility to parasite death, (2) tegumental damage in the form of blistering, (3) reduction of egg production and increased production of abnormally shaped eggs, (4) disorganization of gonadal tissue structures, and (5) a reduction of cell division activities of neoblasts and gonadal stem cells, which was paralleled by reduced transcript levels of stem-cell marker genes.

### Pleiotropic effects of harmonine

Antischistosomal compounds, whether synthetic or natural, often induce alterations in vitality and motility of the adult worms, or in their reproductive fitness (disruption of mating, diminished egg production), in the integrity of the protective tegument, or in the functionality of the parasite nervous system. Remarkably, we found that harmonine affected not just one or few, but all of these parameters simultaneously.

Our *in vitro* studies showed that harmonine reduced the motility of adult worms in a dose-dependent manner, with an EC50 of 8.8 μM and lethality at 10–20 μM within 3 days of culture. These effective concentrations are desirably low, also compared to other natural compounds with described schistosomicial activity, many of which were found to be active in the range of 50–100 μM and even higher [2]. Motor activity alterations belong to the most important indicators of schistosomicidal activity. If the neuromuscular system is affected, mating may be disrupted because the female is released from the gynaecophoric canal of the male partner which eventually leads to degeneration of the female and a complete cessation of egg production [47]. In addition, intact muscle function is required for the suckers to allow the worm to attach to the host endothelial wall, and for functionality of the digestive, reproductive and excretory organs which are lined by musculature [48]. Indeed, harmonine-treated male and female worms separated from each other, were unable to attach with their suckers to the culture well, and stopped egg production at 10 μM. *In vivo*, reduced motor activities would very likely result in removal and degradation of worms by the host and, because egg production is affected, to reduced pathology and the interruption of transmission.

Harmonine also caused adverse effects on the tegument at concentrations as low as 5 μM, which plays crucial roles for nutrient uptake, secretion, osmoregulation, and immune evasion [49]. Damage of the tegument could facilitate the penetration of schistosomicide compounds but also of antibodies to deeper-lying tissues, which may culminate in greater damage to the parasite including disruption of the above-mentioned physiological processes and the ultimate elimination of worms [50, 51]. The tegument is an important target structure for drug discovery, and consequently, compounds affecting the tegument were found to make the parasites more sensitive to the host immune response *in vivo* [52]. Tegumental damage is also induced by PZQ, which may contribute to the compound’s efficacy [17]. Previous work showed the induction of early necrosis in *Leishmania* parasites, which involved the loss of cell-membrane integrity [12]. Also in *S*. *mansoni*, induction of necrosis might contribute to the detrimental effects of harmonine.

### AChE as possible target

Besides the tegument, the nervous system of helminths has been considered a promising target for drug discovery. Several components of the neuronal system are targets of currently approved anthelminthics, including monepantel, levamisole and pyrantel [35, 53, 54]. We found a reduction of schistosomal AChE activity and of AChE transcript levels by harmonine, which suggests two molecular mechanisms that contribute to the observed motility reduction and subsequent paralysis of the parasite’s musculature. We conclude that the reduced enzymatic activity in schistosome protein extracts is not exclusively a consequence of reduced gene transcription, because worms used to prepare extracts have not been treated with harmonine, instead harmonine was added directly to the enzyme assay. According to diverse studies in *S*. *mansoni*, *S*. *haematobium*, *S*. *japonicum*, and S. *bovis*, AChE fulfills two different functions: a classical role in the neuromuscular system for motor activity, and a non-classical role in the tegument related to glucose import from host blood [37, 55]. The tegumental damage observed in harmonine-treated worms might be correlated with the high tegumental expression of AChE [38, 56, 57], while the observed paralysis might be linked to the neuromuscular role. At cholinergic synapses, the neurotransmitter ACh binds to nicotinic ACh receptors (AChRs) and thereby mediates muscular contraction via membrane depolarization. AChE plays a central role in the termination of transmission by hydrolysis of ACh. Treatment of adult schistosomes with either ACh agonists or inhibition of AChE (resulting in higher levels of ACh) led to excessive stimulation, desensitization, and flaccid paralysis of worms [56, 58]. If harmonine inhibits AChE, this should likewise induce paralysis. Indeed, this was observed in our study. To clarify whether the antischistosomal effects of harmonine are mainly due to targeting of AChE, a gradual knock-down of AChE expression to a similar degree as found for the reduction of AChE activity by harmonine might be of interest for future work. While we found a generally high expression and enzymatic activity of AChE in males and unpaired females, both parameters were strongly decreased in paired females. This might reflect a reduced need in motor activity of the female after pairing and/or a different need for AChE-mediated glucose uptake. Up-and down-regulation of genes in females after pairing and separation, respectively, is a known phenomenon [59, 60]. We also found other genes involved in the function of the neuromuscular synapse being downregulated, such as genes annotated as nicotinic AChR (Smp_176310, Smp_139330, Smp_180570) and as choline acetyltransferase (Smp_146910), the enzyme involved in ACh synthesis [39]. Altogether, it seems likely this contributes to a reduced motor activity of females after pairing. Our findings of a sex- and pairing-dependent AChE expression and activity pattern adds novel aspects to the wealth of data on AChE function in schistosome biology, and might explain why females appeared less sensitive than males towards harmonine during the first 24 h of treatment.

The druggability of AChE was demonstrated by the use of the AChE inhibitor metrifonate against *S*. *haematobium* in the past [35]. However, metrifonate was withdrawn from the market because of the need for multiple doses, its toxicity to the host, and its unsatisfying efficacy against other schistosome species [61]. Nonetheless, AChE remains an interesting antischistosomal target for rational drug design. That species-specific optimization is achievable was for instance demonstrated by re-engineering inhibitors toward higher specificity for *Anopheles* AChE than human AChE [62]. Also for harmonine, rational drug design might be used to obtain even better efficacies.

### Schistosome stem cells as targets

Our conclusion that there are further harmonine targets beyond AChE is based on three observations: (1) the merely weak AChE inhibitory capacity of harmonine, (2) the multifaceted phenotypes observed after treatment with harmonine, and (3) effects by the AChE inhibitor metrifonate that were different in strength involving a rapid paralysis within only 1–3 hours, which was reversible [63]. Additional targets might be related to stem-cell biology. Due to the fundamental role of stem cells in the parasite life cycle and parasite survival, it was recently proposed that many helminth infections may be considered as stem cell diseases [45, 64]. Compounds targeting stem cells are thus particularly attractive candidates for drug development. In order to not only assess but objectively quantify stem cell effects, we established a 3D reconstruction approach of schistosome ovaries and testes to quantify proliferating stem cells. Harmonine significantly reduced the number of proliferating stem cells in gonadal tissues and the parenchyma where they are known as neoblasts [44]. Furthermore, we used for the first time quantification of *nanos* gene expression as a measure for compound-induced effects. *Nanos-1* was characterized as a germline-specific stem cell marker in adult *S*. *mansoni* [42, 65] and is known as a conserved regulator of germ cell development [43]. Somatic stem cells, so-called neoblasts, were only recently identified in adult schistosomes and are characterized by expression of *nanos-1* and *nanos-2* [44]. *Nanos-2* is a post-transcriptional regulator responsible for the formation, development, and maintenance of pluripotent cells in many metazoans [66]. Knock-down of *nanos-1* and *nanos-2* in *S*. *mansoni* resulted in loss of mature germ cells and in degenerated testes, and it was concluded that *nanos-1* and *nanos-2* are required for germ-cell differentiation [42]. Since the levels of *nanos* expression were reduced upon harmonine treatment, one could speculate about a reduction of total stem cell numbers by induction of cell death. However, it is not clear whether *nanos* expression might also be affected in alive but cell cycle-arrested stem cells. Therefore, it remains open whether harmonine induces cell-cycle arrest or cell death in gonadal and somatic stem cells. In the case of cell-cycle arrest, removal of harmonine before EdU addition to the culture would lead to a resumption of cell proliferation and may clarify this question.

AChE as the target of harmonine in schistosomal stem cells is another attractive hypothesis. Indeed, according to previous RNAseq analyses, transcripts of a variety of ACh receptors were found in ovaries and testes of *S*. *mansoni* [39], suggesting a non-neuronal role of the AChE-ACh-AChR axis in gonads. In addition, AChE is known to be expressed in certain stem cells where it regulates cell proliferation. For instance, embryonic stem cells of human and mouse express AChE and showed reduced proliferation upon ACh stimulation [67, 68], just like an ACh over-abundance by inhibition of AChE activity might do. Furthermore, inhibition of AChE by organophosphates in mesenchymal stem cells or by donezepil in neural stem cells led to suppression of proliferation, differentiation and self-renewal ability [41, 69]. Therefore, future studies should address the possible role of ACh signaling in schistosomal stem cells in detail, and we propose RT-qPCR analysis of sorted stem cells as a start. This might reveal new insights into AChE function in schistosomes and aid in developing novel therapies.

As a side note, it appears biologically astonishing how *H*. *axyridis* is able to resist the toxic potential of harmonine which accumulates in the hemolymph up to 7 μg per mg body mass [5]. This resistance is even more remarkable seeing the potential of harmonine to inhibit such crucial processes like stem cell proliferation, and will fuel the curiosity in future research.

### Insects as source for antischistosomal compounds

Alkaloids are natural compounds that are widespread in plants, bacteria, fungi, and animals. *In vitro* schistosomicidal activity was demonstrated for several plant-derived alkaloids, such as piplartine from *Piper* species [2, 3]. Harmonine is the first animal-derived alkaloid found to have antischistosomal capacity. Notably, the chemical structure of the alkaloid harmonine, an aliphatic long-chain diamine, is quite different to these other active alkaloids.

To date, only very few other studies addressed animal-derived compounds and their antischistosomal activities. These are compounds from sea cucumbers, skin secretions of a South-American tree-frog species, and undefined compounds from snake venom [3, 70–72]. From insects, so far only two complex product mixtures, but no defined compounds, were described to have direct or indirect anthelminthic effects. Propolis (“bee glue”) is a complex resinous bee hive product and was shown to have various anti-protozoal [73], fungicidal, and antimicrobial properties [74]. Furthermore, *in vivo* treatment of *S*. *mansoni*-infected mice with propolis produced by *Apis mellifera* reduced the worm burden but increased granuloma diameter [4], which pointed to an immune-modulatory effect rather than a direct effect on worm vitality. Unfortunately, there are no data in the literature on *in vitro*-culture experiments that demonstrate any direct effects of propolis or propolis-derived compounds on schistosomes. The second anthelmintic insect-derived product described in literature is bee venom. Bee venom is a complex mixture of enzymes, biogenic amines and peptides with described anti-inflammatory capacity and has been studied as an alternative medicine for inflammatory diseases and cancer [75, 76]. As with propolis, a reduction of worm burden was found upon treatment of *S*. *mansoni*-infected mice [4], but unfortunately without analysis of direct effects, mode of action or possible schistosomal targets. Thus harmonine and its effects on AChE activity and stem cell gene expression provides the very first insight into possible mechanisms of action of an insect-derived compound.

### Outlook

In view of the broad *in vitro* activity of harmonine found against microbes [7, 10], protozoans [7, 12] and helminths (this study), it is tempting to promote this insect-derived compound as a novel universal weapon, a kind of swiss-army knife against multiple pathogens. However, we found first evidence of cytotoxicity against HepG2 cells at concentrations of 50 μM and higher, indicating that harmonine in its current form is not yet an ideal lead candidate, but certainly a valuable basis for structure/activity-based compound development. The EC50 of 8.8 μM obtained for the reduction of schistosome motility by harmonine is already a promising start, as this concentration did not lead to any cytotoxic effects against HepG2 cells. In summary, this study provided clear evidence for the antischistosomal activity of the lady beetle-derived compound harmonine together with biologically highly interesting effects on AChE activity, inhibition of stem-cell proliferation and gene expression. This is the first time to proof a direct effect of a defined insect-derived compound on schistosomes, and harmonine may serve as basis for the development of new antischistosomal, or even broader antiparasitic compounds. This study highlights the potential of exploiting insects for the discovery of anthelminthics and motivates the screening of insect compound libraries for novel anthelminthic compounds in the future.

## Supporting information

S1 Fig Carboxyl-esterase domains in AChE orthologs of *S. mansoni*. PFAM domains were revealed by the online-tool SMART (http://smart.embl-heidelberg.de/).
(TIF)

S2 Fig Characterization of AChE activity. **(A-D)** Inhibition of enzymatic activity by the AChE inhibitor physostigmine. Enzymatic activity over time of AChE from electric eel (A) or of protein lysates of paired *S*. *mansoni* males (B) after adding different concentrations of physostigmine (0 – 2500 nM). One representative out of two similar experiments is shown. Relative AChE activity of electric eel (C) and schistosome lysate (D) at 30 min after adding different concentrations of physostigmine, with the activity at 0 μM set as 100%. Mean values of two experiments were used. IC50 was calculated by non-linear least squares curve fitting using the ic50.tk tool. **(E)** Michaelis-Menten plot of the harmonine effect on AChE activity. AChE from the model organism *E*. *electricus* was co-incubated with 10 μM or 50 μM harmonine and increasing concentrations of the substrate (S) acetylthiocholine (up to 2.2 mM). In the control, the solvent DMSO without harmonine was added. The reaction velocity (v) describes substrate conversion in μmol/min using AChE at a constant concentration of 0.12 nM. Data points are based on technical replicates, error bars are SEM values. Assay buffer: 38 mM Tris-HCl, pH 8.0, 100 mM NaCl, 20 mM MgCl2, 330 mM 5,5′-Dithiobis(2-nitrobenzoic acid) [DTNB]. Reactions were performed in 96-well plates (206 μl/well) and absorption changes at 410 nm were recorded for 3 min; the initial slopes of absorption over time plots were used to calculate reaction velocities according to the Beer-Lambert law.
(TIF)

S3 Fig Effect of harmonine on proliferation of gonadal stem cells (conventional CLSM without IMARIS processing). Worms were treated with 5 μM (C, D) or 10 μM (E, F) harmonine or with an equivalent amount of solvent as negative control (A, B) for 72 h, with EdU added for the last 24 h. EdU-positive proliferating stem cells in female ovaries (A, C, E) or male testes (B, D, F). Stem cells are mainly found in the immature (iO) part of the ovary, not in the mature (mO) part. One z-plane of one representative female or male per condition from two experiments is shown. Scale bar: 35 μm.
(TIF)

S1 Movie 3D animation of a female ovary stained for EdU-positive proliferating stem cells and Hoechst-positive cells. Still images are shown in Fig 7.
(MPG)
