# Supplementary figures and images for: Insects in anthelminthics research: Lady beetle-derived harmonine affects survival, reproduction and stem cell proliferation of Schistosoma mansoni
Source: PLoS Negl Trop Dis. 2019 Mar 14;13(3):e0007240. doi: 10.1371/journal.pntd.0007240 (PMC6436750; doi:10.1371/journal.pntd.0007240)

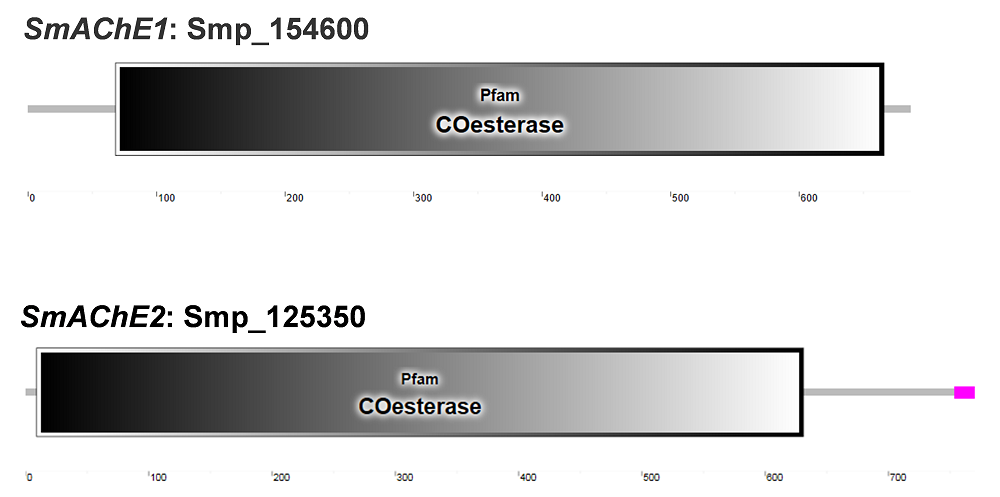

Supplement: S1 Fig — PFAM domains were revealed by the online-tool SMART (http://smart.embl-heidelberg.de/). (TIF) [file pntd.0007240.s001.tif]

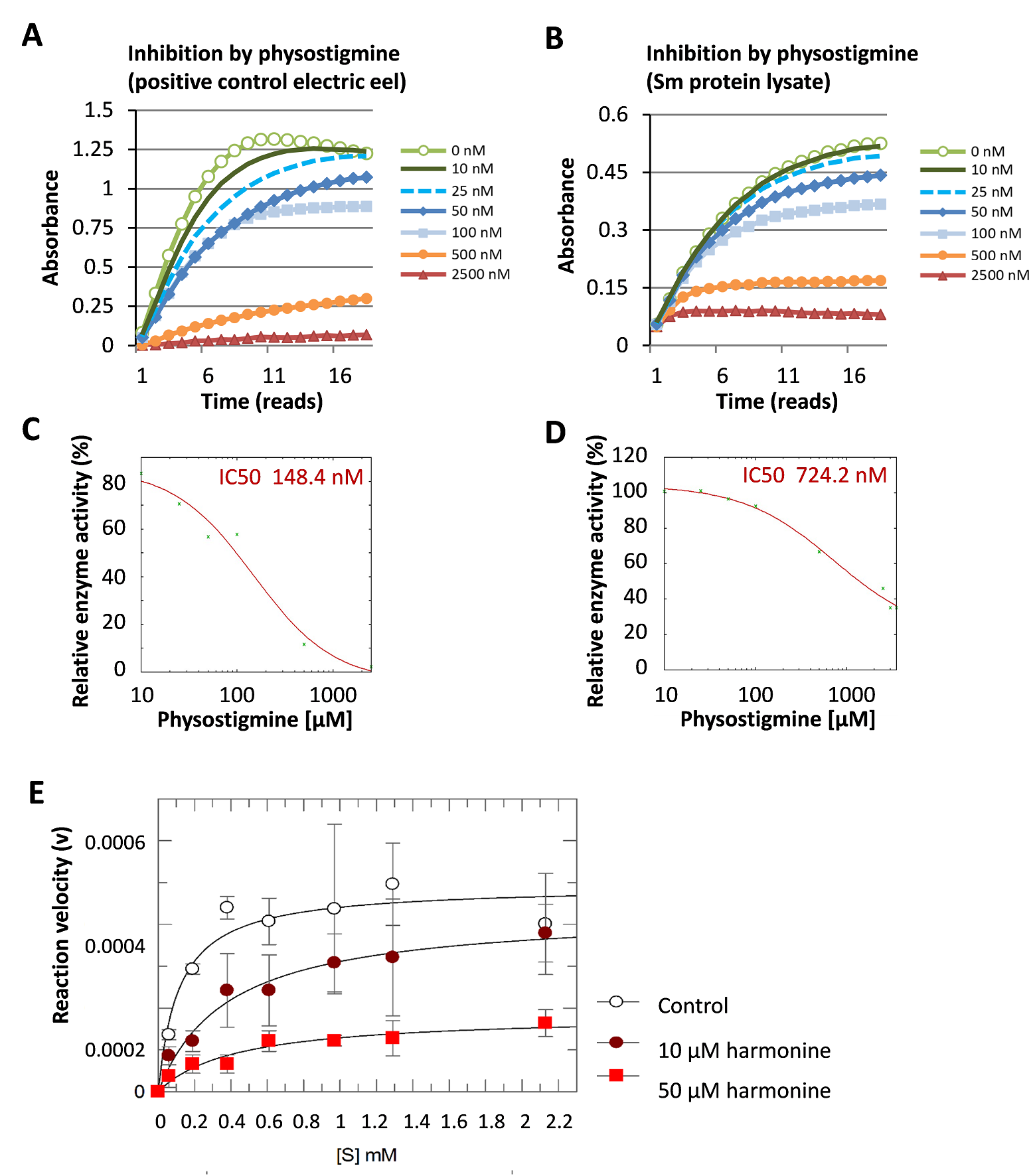

Supplement: S2 Fig — (A-D) Inhibition of enzymatic activity by the AChE inhibitor physostigmine. Enzymatic activity over time of AChE from electric eel (A) or of protein lysates of paired S. mansoni males (B) after adding different concentrations of physostigmine (0 – 2500 nM). One representative out of two similar experiments is shown. Relative AChE activity of electric eel (C) and schistosome lysate (D) at 30 min after adding different concentrations of physostigmine, with the activity at 0 μM set as 100%. Mean values of two experiments were used. IC50 was calculated by non-linear least squares curve fitting using the ic50.tk tool. (E) Michaelis-Menten plot of the harmonine effect on AChE activity. AChE from the model organism E. electricus was co-incubated with 10 μM or 50 μM harmonine and increasing concentrations of the substrate (S) acetylthiocholine (up to 2.2 mM). In the control, the solvent DMSO without harmonine was added. The reaction velocity (v) describes substrate conversion in μmol/min using AChE at a constant concentration of 0.12 nM. Data points are based on technical replicates, error bars are SEM values. Assay buffer: 38 mM Tris-HCl, pH 8.0, 100 mM NaCl, 20 mM MgCl2, 330 mM 5,5′-Dithiobis(2-nitrobenzoic acid) [DTNB]. Reactions were performed in 96-well plates (206 μl/well) and absorption changes at 410 nm were recorded for 3 min; the initial slopes of absorption over time plots were used to calculate reaction velocities according to the Beer-Lambert law. (TIF) [file pntd.0007240.s002.tif]

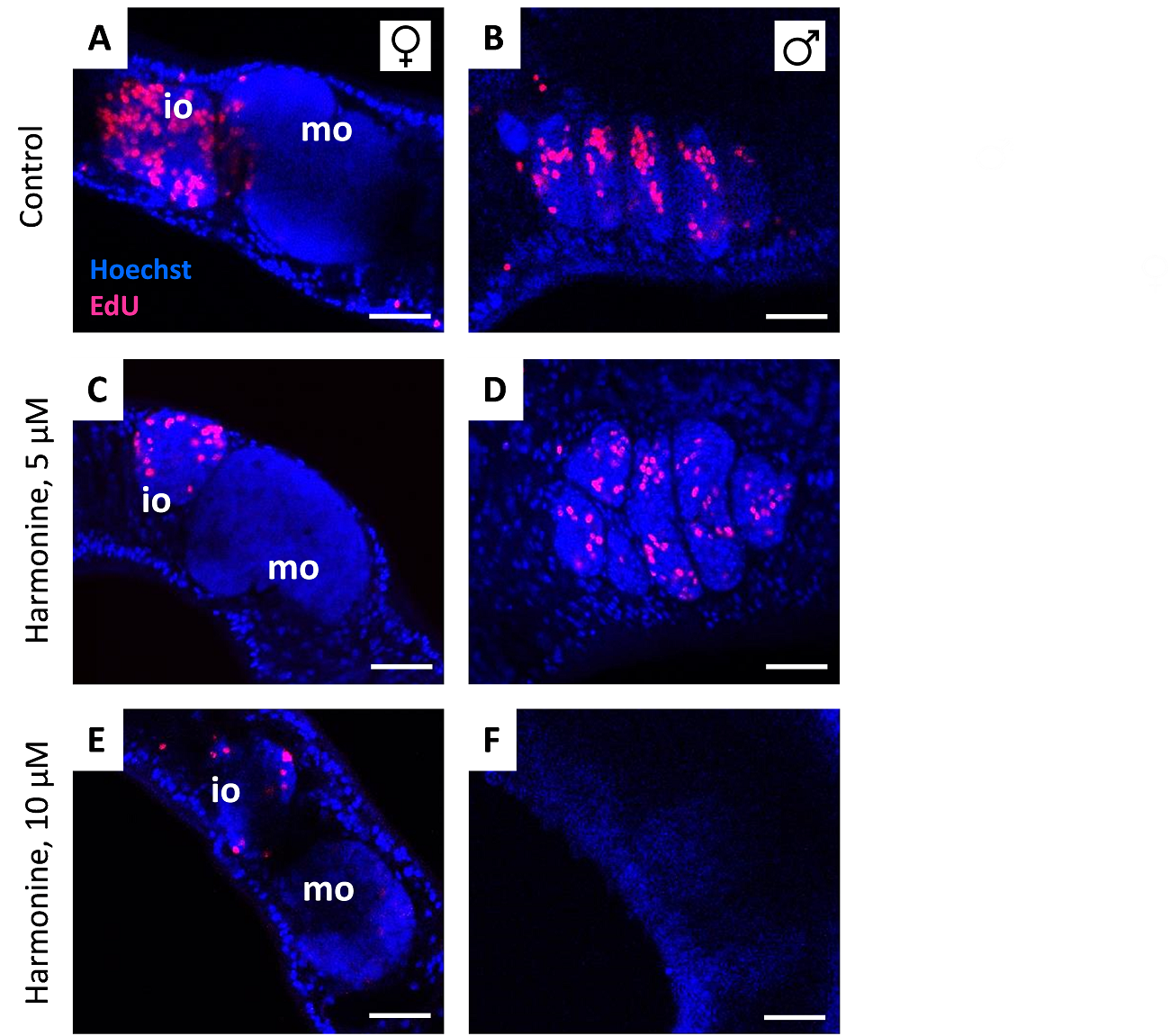

Supplement: S3 Fig — Worms were treated with 5 μM (C, D) or 10 μM (E, F) harmonine or with an equivalent amount of solvent as negative control (A, B) for 72 h, with EdU added for the last 24 h. EdU-positive proliferating stem cells in female ovaries (A, C, E) or male testes (B, D, F). Stem cells are mainly found in the immature (iO) part of the ovary, not in the mature (mO) part. One z-plane of one representative female or male per condition from two experiments is shown. Scale bar: 35 μm. (TIF) [file pntd.0007240.s003.tif]
